# Death

**Published:** 1890-04-19

**Authors:** 


### Words of Consolation.

### DEATH.

If we look round us in the world we cannot but see that old and young, rich and poor, wise and simple, alike go to that bourne from which no traveller returns. One event happeneth to all men, and, since death is inevitable, surely the loving Father of mankind has provided some solace for those fainting souls who are failing for fear as the time of their departure draws nigh.

Yes, the Gospel of Love has comfort for these sorrowing hearts. The Blessed Master, whose whole earthly life was overshadowed by the darkness of His coming agony, has left messages for them.

The night before His crucifixion He hushed to rest the murmurs, the questionings, the strife of His anxious and bewildered followers. His words fell calm and clear on their ears, Let not your heart be troubled: ye believe in God, believe also in me. In my Father's house are many mansions. If it were not so, I would have told you. I go to prepare a place for you: and if I go to prepare a place for you, I will come again and receive you unto myself. He came again to His Apostles in unexpected ways, and took them to Himself by fire, by sword, by persecutions of all sorts. He came to them in the still small voice of comfort and in the sacrament of His love. He was with them spiritually in life and death years after His bodily presence had ascended into Heaven.

And doubt not, oh! watching, fearful soul, that the promise is also for thee. It is as much thine as the Apostles', to whom it was first given, and through whom it has descended to the whole Church which the Lord shalt gather together at His second coming. Thou wert not forgotten, humble and unworthy as thou mayest deem thyself.

On thee and thine, thy warfare and thine end,
Even in His hour of agony He thought.

If thou art one of those who have believed on Jesus through the words of His Apostles, so surely thou shalt inherit the promise: Blessed are they that have not seen and yet have believed.

He has not said how or when He will come to thee; thou must wait His time; thou must tarry till He come. He will not leave thee comfortless in the last dread hour. He will come for thee Himself. When thou comest to the waters He will be with thee, and bear thee through the valley of the shadow of death, for He has trodden the path as man, and knows all its terrors. It may be that this last journey, so dreaded by thee in life, will be the sweetest thou hast ever taken, for He will come and receive thee unto Himself, that where He is thou mayest be also. O glorious promise! O blessed ending to a life of sorrow and sadness! To be with Him; to be ever with the Lord! What joy, what bliss to dwell in the courts of the King's palace, to be with Him, our Saviour, in Paradise. Fear not, then. When He comes for thee, lean hard upon His Hand. Though the outward man perish, yet the inward man is renewed day by day; if thou art absent from the body thou wilt be present with the Lord. "The veilèd Guest in the starlight dim" will speak deep words of grace to thee, and as thou claspest the Master's Hand thou wilt grow more rapt and joyful, and before thou knowest thou wilt find thyself in the Holy Land. Rest, then, in calmness and quietness for thy call. Let thy prayer be ever, "Lord, come when Thou wilt and as Thou wilt, only let me receive Thee without sin or shame," and "Suffer me not at my last hour for any pains of death to fall from Thee."

